# Supplementary material for: Structure of the human plasma membrane Ca2+-ATPase 1 in complex with its obligatory subunit neuroplastin
Source: Nat Commun. 2018 Sep 6;9:3623. doi: 10.1038/s41467-018-06075-7 (PMC6127144; doi:10.1038/s41467-018-06075-7)
Supplement: Supplementary file 1 — Supplementary Information [file 41467_2018_6075_MOESM1_ESM.pdf]

Structure of the human plasma membrane Ca^{2+}-ATPase 1 in complex with its obligatory subunit neuroplastin

Gong et al.

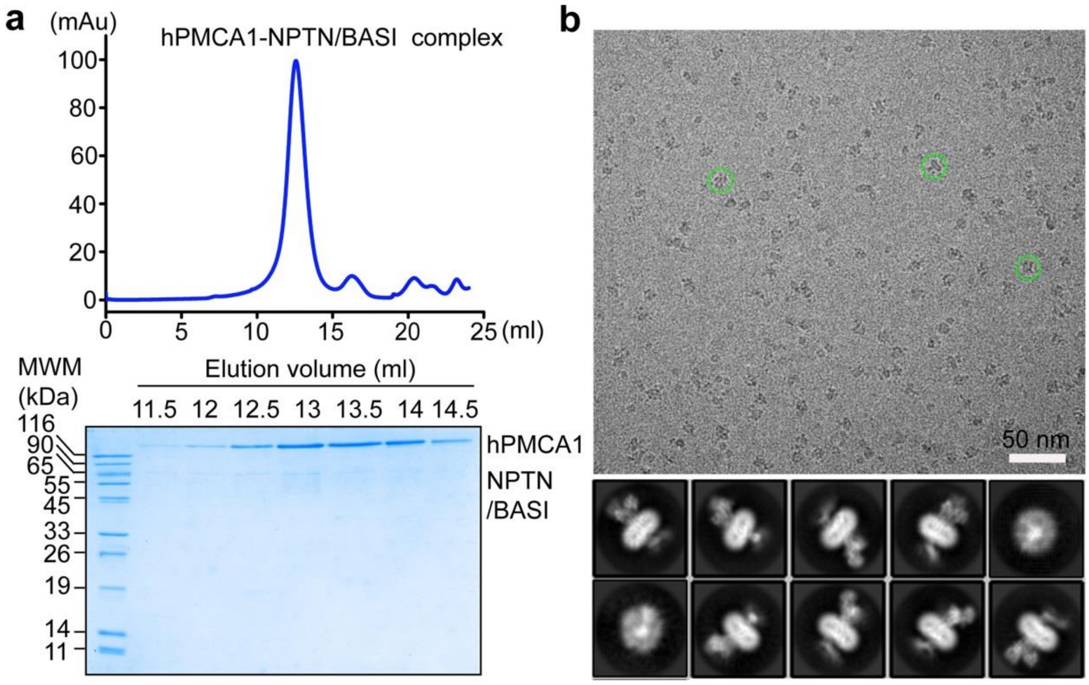

Supplementary Figure 1 Cryo-EM analysis of the hPMCA1-NPTN complex. a, The hPMCA1-NPTN/BASI complexes were purified by Superose-6 column. The indicated fractions were subjected to SDS-PAGE, and the proteins were visualized by Coomassie blue staining. MS analysis of the additional poorly resolved bands identified both NPTN and BASI. hPMCA1-NPTN/BASI represents a mixture of the hPMCA1-NPTN complex and the hPMCA1-BASI complex. **b,** Representative cryo-EM micrograph and 2D class averages.

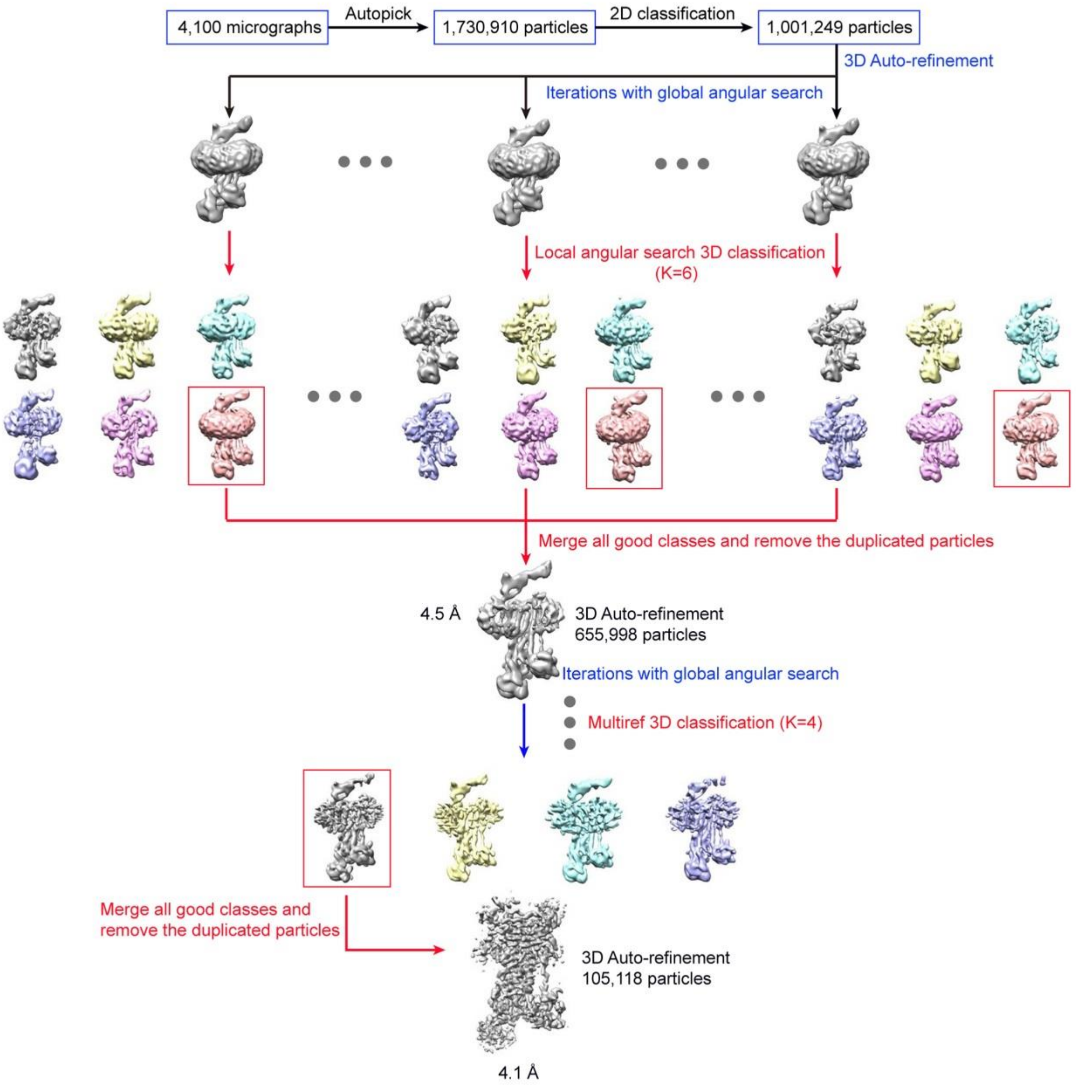

Supplementary Figure 2 Flowchart for cryo-EM data processing. Please refer to Image Processing in the Methods section for details of the flowchart.

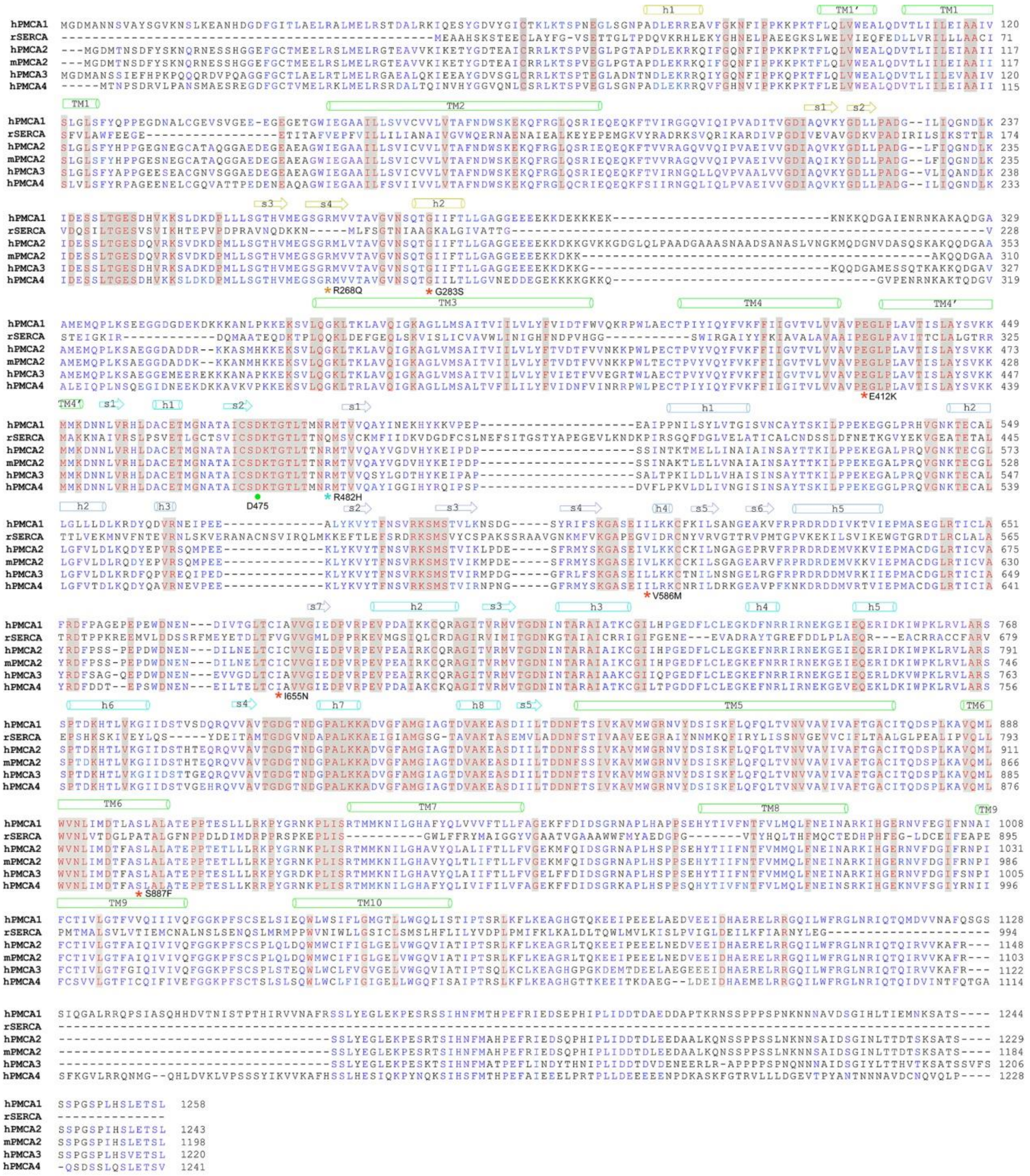

Supplementary Figure 3 Sequence alignment of hPMCA1 with its homologues. The sequences were aligned using ClustalW. The secondary structural elements of hPMCA1 are indicated above the sequence alignment and are colour coded using the same colour scheme used in Fig. 1c for the overall structure. r: rabbit; m: mouse. The pathogenic residues are labelled with a star.

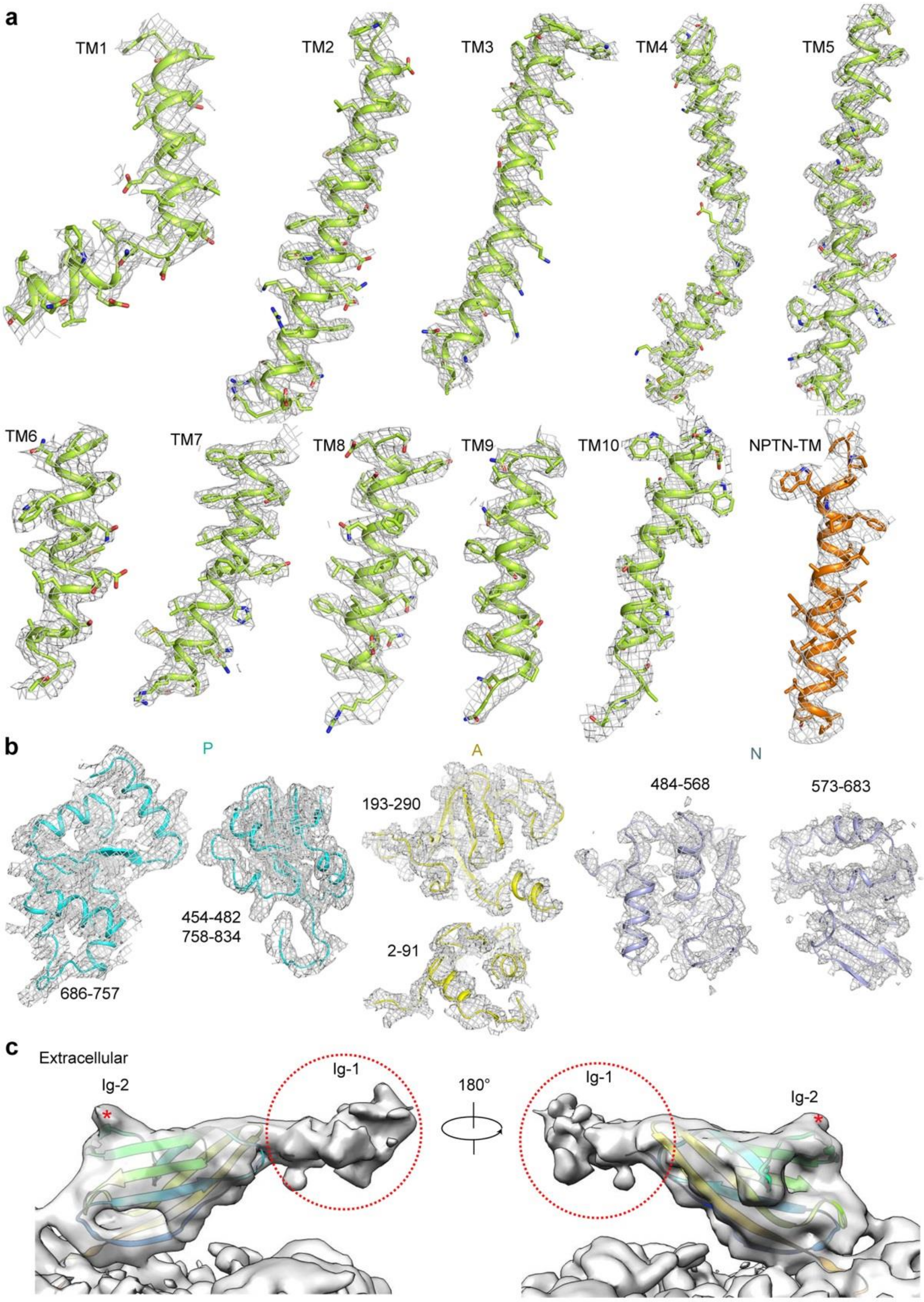

Supplementary Figure 4 EM maps of representative segments of hPMCA1-NPTN. a, EM maps of each TM helix. **b,** EM maps of the cytoplasmic domains. **c,** Crystal structure of Ig-2 (PDB: 2WV3) docked into the low-pass-filtered 6.0-Å EM map, which is shown at a contour level of 0.019. The red star represents

glycosylation modification. The density of Ig-1 is not sufficient to show the docking.

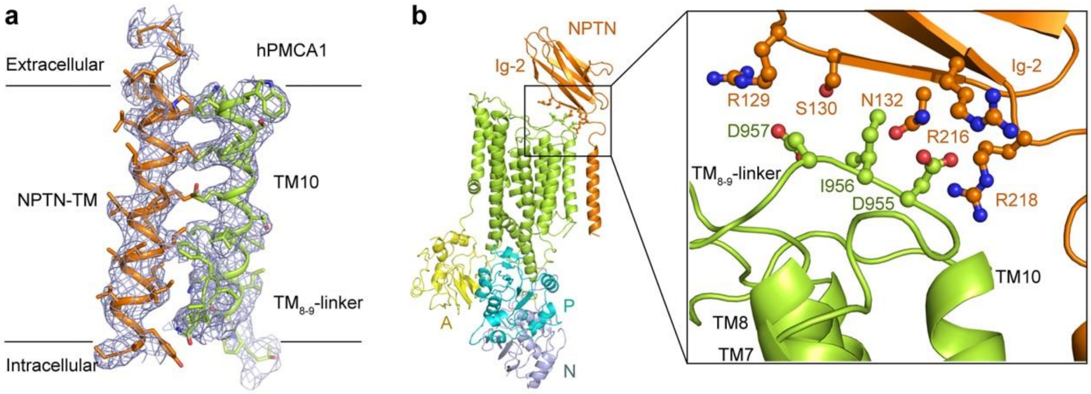

Supplementary Figure 5 Interactions between the NPTN and hPMCA1. a, The EM map of the NPTN-TM, TM10, and TM_{8-9}-linker is shown. **b,** The residues that may mediate the interactions between Ig-2 and the TM_{7-8}-linker are highlighted.

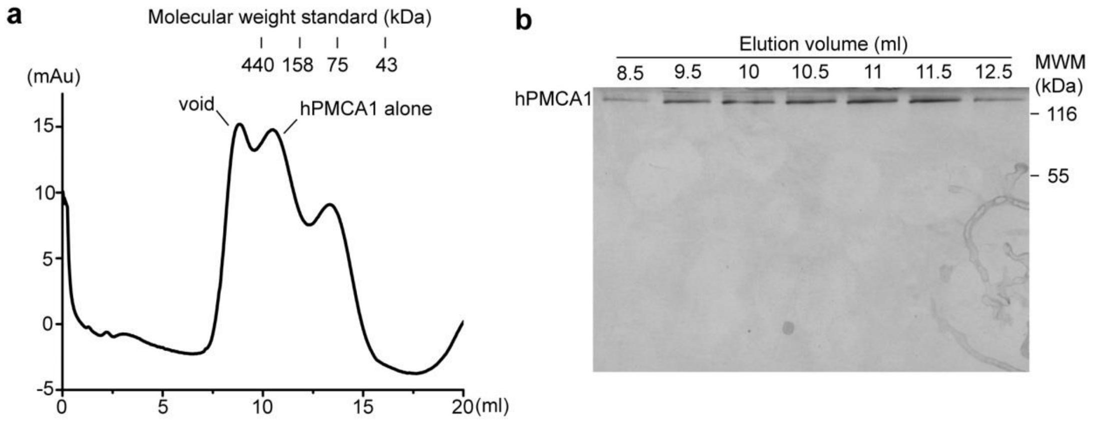

Supplementary Figure 6 Purification of the hPMCA1 alone proteins. a, The hPMCA1 alone proteins were purified by Superdex 200 column. The proteins with an elution volume of 11 ml were used for activity assay. **b,** The indicated fractions were subjected to SDS-PAGE, and the proteins were visualized by Coomassie blue staining.

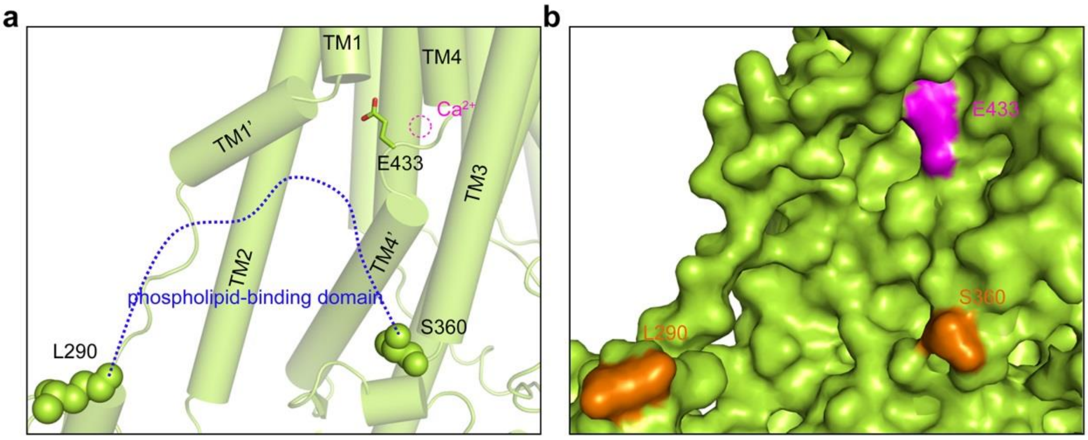

Supplementary Figure 7 The location of the phospholipid-binding domain. a, The phospholipid-binding domain is located in the vicinity of the large cytosolic vestibule of Ca^{2+} permeation pathway. **b,** The molecular surface of panel a.

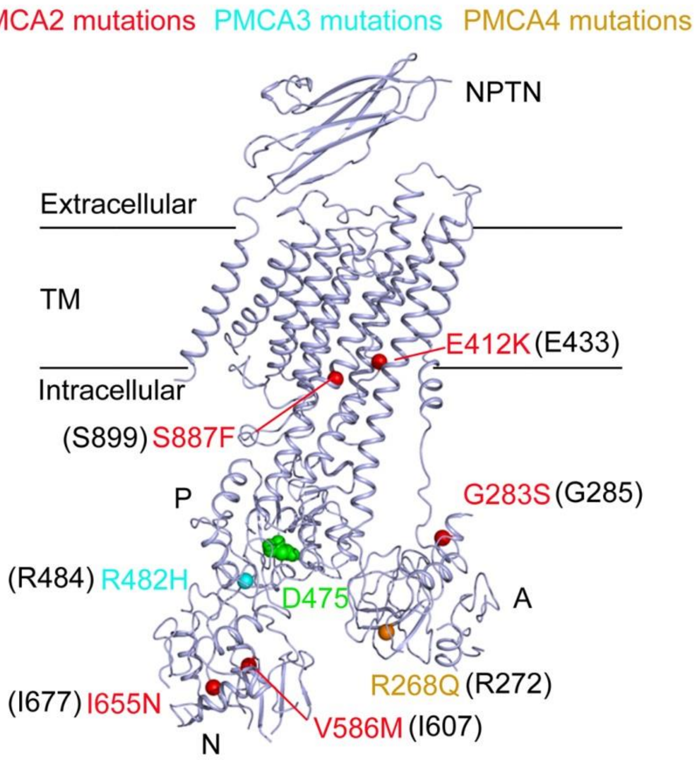

Supplementary Figure 8 Mapping of the disease-associated point mutations onto the structure of hPMCA1. The Ca atoms of the pathogenic residues are shown as spheres. The phosphorylation site D475 are shown as spheres. The corresponding residues on hPMCA1 are shown in the black parenthesis.

Supplementary Table 1 Mass spectrometric analyses of the additional bands

Description	Unique peptides	Coverage
BASI_HUMAN	22	45.97%
NPTN_HUMAN	19	38.19%

Supplementary Table 2 Summary of data collection and model statistics

Items	Parameters
Data collection	
EM equipment	FEI Titan Krios
Voltage (kV)	300
Detector	Gatan K2 Summit
Pixel size (Å)	1.091
Electron dose (e^-/Å^2)	50
Defocus range (μm)	-1.5 to -2.5
Reconstruction	
Software	RELION 2.0
Maps	hPMCA1-NPTN
Number of used Particles	105,118
Symmetry	C1
Final resolution (Å)	4.1 Å
Map sharpening B-factor (Å^2)	-200
Model building	
Software	Coot
Refinement	
Software	Phenix
Validation	
R.m.s deviations	
Bonds length (Å)	0.01
Bonds length (Å)	1.42
Ramachandran plot statistics (%)	
Preferred	86.6 %
Allowed	13.3 %
Outlier	0.1 %

Supplementary Table 3 The primers for vector construction

Primers (5'-3')	
PM1-NotI-F	ATTATGCGGCCGCATGGGCGACATGGCTAATAAC
PM1-XhoI-R	ATATGCTCGAGCAGGGAGGTTTCTAAGGAGTGCAG
